# Pre-transplant *FLT3/ITD* status predicts outcome in FLT3-mutated acute myeloid leukemia following allogeneic stem cell transplantation

**DOI:** 10.1007/s00277-020-04026-1

**Published:** 2020-04-24

**Authors:** Grzegorz Helbig, Anna Koclęga, Agata Wieczorkiewicz-Kabut, Krzysztof Woźniczka, Anna Kopińska, Kinga Boral, Iwona Grygoruk-Wiśniowska, Małgorzata Stachowicz, Agnieszka Karolczyk

**Affiliations:** Department of Hematology and Bone Marrow Transplantation, School of Medicine in Katowice, Medical University of Silesia, Dąbrowski street 25, 40-032 Katowice, Poland

**Keywords:** Acute myeloid leukemia, FLT3/ITD mutation, Allogeneic stem cell transplantation, Overall survival, Leukemia-free survival, Minimal residual disease

## Abstract

Acute myeloid leukemia (AML) with fetal liver tyrosine kinase 3 (FLT3) internal tandem duplication (ITD) is associated with poor prognosis, and allogeneic stem cell transplantation (Allo-SCT) seems to be the preferred therapeutic approach. However, the predictors of post-transplant outcomes were not well-defined. The aim of the study was to evaluate the significance of *FLT3/ITD* mutation by polymerase chain reaction as minimal residual disease (MRD) marker of outcomes after transplantation. We identified 43 patients (28 females and 15 males) with FLT3-mutated AML at the median age of 45 years who were allografted between 2009 and 2019. Hematological status at transplant was as follows: the first complete remission (CR1) in 29 patients, CR2 in 5, and 9 patients were transplanted in marrow aplasia (MA). Twenty-seven patients were FLT3 MRD negative at transplant. Median time from diagnosis to transplant was 16.7 months. Post-allograft CR rate was 88%. The relapse incidence (RI) was lower for patients who were FLT3 MRD negative at transplant when compared with those with FLT3 MRD positivity (41% vs 59%; *p* = 0.01). The patients who eradicated *FLT3/ITD* at day + 30 after transplant had lower RI than those with detectable *FLT3/ITD* (23% vs 76%; *p* = <0.001). The 2-year LFS and OS were 53% and 54%, with the median OS and LFS of 28 months and 27 months, respectively. Patients with CR1/2 and FLT3 MRD(−) had a 2-year OS of 80%. The FLT3 MRD negativity at transplant prolonged LFS in multivariate analysis (HR 5.3 95%CI 1.97–14.2); *p* < 0.001), whereas FLT3 MRD negativity and unrelated donor predicted favorable OS.

## Introduction

The FMS-like tyrosine kinase-3 (FLT3) is expressed by myeloid and lymphoid progenitors, and it is responsible for differentiation, proliferation, and apoptosis of the cells. Internal tandem duplication in the juxtamembrane domain of FLT3 (FLT3/ITD) is frequent molecular aberration which is detected in approximately 30% of the patients with acute myeloid leukemia and normal diploid karyotype (AML-CN). This mutation leads to ligand-independent activation of the receptor and its signaling pathways [1]. Many studies have demonstrated that the occurrence of *FLT3/ITD* mutation in AML remains a poor prognostic factor owing for high relapse rate and shortened survival [2, 3]. However, it was recently shown that this aberration produces a negative effect only if the ratio of mutated to normal alleles is > 0.5 [4].

The role of allogeneic stem cell transplantation (Allo-SCT) in FLT3*-*mutated patients in the first complete remission (CR1) seems to be controversial, especially in the view of the newest findings in the molecular signature of AML. The assessment of *FLT3/ITD* allelic ratio and co-occurrence of NPM1 mutation have a confirmed impact on risk stratification and therapeutic approaches [4–6]. The results from the donor versus no donor study of adult patients with *FLT3/ITD* mutation treated according to the United Kingdom Medical Research Council (UK MRC) AML protocol have demonstrated lower relapse rate for patients with donor, but no difference in overall survivals (OS) between compared groups [7]. It was also found that FLT3-mutated patients when compared with FLT3-unmutated had higher relapse incidence (RI) and lower leukemia-free survival (LFS) even after Allo-SCT. Nevertheless, more than 50% of the transplanted patients carrying this mutation were leukemia free at 2 years. Of note is that other predictors than *FLT3/ITD* mutation may play a role in leukemia relapse after transplantation [8]. The *FLT3/ITD* mutation may serve as a marker of minimal residual disease (MRD), and its utility has been evaluated in a single study so far [9].

The aim of the study was to evaluate the potential factors which may have an impact on a post-transplant outcome in FLT3-mutated patients with AML following Allo-SCT.

## Material and methods

The study patients were retrospectively identified through the use of our institutional database of medical records. The diagnosis of AML and response criteria to therapy were based on European Leukemia Net Recommendations [4]. Marrow aplasia (MA) was defined as pancytopenia with bone marrow aplasia after salvage chemotherapy. Patients were treated according to Polish Adult Leukemia Group (PALG) protocol [www.palg.witaj.pl] and induction chemotherapy included DA+/− C regimen (daunorubicin, cytarabine, cladribine). For those who achieved complete remission, 2 cycles of consolidative chemotherapy consisting of mitoxantrone with cytarabine (HAM) or cytarabine alone (ARA-C) were administered. For patients with resistant disease (RD) after 2 inductions, 3 different salvage regimens were given according to the treating physician: CLAM (cladribine, cytarabine, mitoxantrone), MEC (mitoxantrone, etoposide, cytarabine), and FLAG-IDA (fludarabine, cytarabine, idarubicin, G-CSF). All patients who were FLT3-mutated at diagnosis were proceeded to Allo-SCT according to the center policy.

The *FLT3/ITD* and nucleophosmin (NPM1) mutation analysis were performed at diagnosis, and FLT3/ITD was then performed before conditioning commencement and at day + 30 after procedure. Multiplex fluorescence-based PCR method was performed on DNA isolated from bone marrow aspirate sample as described elsewhere. The sensitivity of this assay is approximately 5% [10]. All patients were grouped according to the molecular and cytogenetic genetic stratification of the European Leukemia Net (ELN) 2010 [11] as most AML patients (38/43) were diagnosed < 2017. Data on *FLT3/ITD* allelic ratio was available only for 4 patients, and therefore this parameter was not analyzed. Chimerism of unseparated blood leukocytes was assessed by a short tandem repeat polymerase chain reaction.

### Statistics

Time to event was assessed from the day of transplantation. Nonparametric comparisons of group means were performed by using the Mann-Whitney *U* test. Proportions were compared by Fisher exact test. The Kruskal-Wallis test was used to compare more than two independent groups of variables. Non-relapse mortality (NRM) was defined as all deaths before disease recurrence. The distribution for overall survival (OS) and leukemia-free survival (LFS) was estimated using the method of Kaplan and Meier and compared using the log-rank test. A *p* value less than 0.05 was considered significant. Proportional hazards models (Cox regression) were fitted to investigate effects of prognostic factors for OS.A Spearman’s rank test was used to assess the correlation between variables. All computations were performed with StatSoft Poland analysis software (version 10.0).

## Results

### Patient characteristics

Forty-three patients (28 females and 15 males) with AML at median age of 45 years (range 19–67) underwent Allo-SCT between 2009 and 2019. Four patients were ≥ 60 years at transplant and 3 had prior myelodysplastic syndrome. All patients demonstrated detectable *FLT3/ITD* mutation by polymerase chain reaction (PCR). Sixty percent of the subjects had normal diploid karyotype, and trisomy 8 was the most common cytogenetic abnormality (*n* = 4). Study patients were stratified into the following risk groups: 2 patients were categorized to adverse risk group as they had monosomies of chromosomes 5 and 7; 4 patients were in intermediate-2 group as they had cytogenetic abnormalities not classified as favorable or adverse; and 30 patients were in intermediate-1 group, and in 7 patients, no metaphases were obtained (they were stratified to intermediate-1 category based on molecular status only).

Induction regimen consisted of DAC (*n* = 30) or DA (*n* = 13). No induction death was observed. Thirty patients (70%) achieved the first complete remission (CR1), and CR2 was demonstrated in 4 after re-induction. Nine subjects developed irreversible marrow aplasia after chemotherapy.

Hematological status at transplant was as follows: CR1 in 29 patients, CR2 in 5, and 9 patients were transplanted in MA. Twenty-seven patients had FLT3 MRD negativity before procedure. Median time from diagnosis to transplant was 16.7 months (range 5.1–76). Patients’ characteristics are shown in Table 1.

**Table 1:** Patients characteristics

Variable	*n* = 43
Gender (female/male)	28/15
Age, years; median, range	45 (19–67)
Hemoglobin level (g/dl); median, range	8.8 (5.7–13.0)
Leukocyte count (× 10^9^/l); median, range	47.5 (1.7–197.0)
Platelet count (× 10^9^/l); median, range	66 (5–148)
Blasts in blood (%); median, range	79 (11–100)
Blasts in bone marrow (%); median, range	87 (24–100)
Splenomegaly, *n*; %	12 (28)
Hepatomegaly, *n*; %	7 (16)
Lymphadenopathy, *n*; %	11 (25)
FAB subtype M1 M2 M4 M5	7 15 18 3
Karyotype, *n*; % diploid + 8 − 5q + 11 inv9 − 7q t(1;7) + 3,+ 13 no metaphases	26 (60) 4 (9) 1 (2) 1 (2) 1 (2) 1 (2) 1 (2) 1 (2) 7 (19)
Prior MDS, *n*; %	3 (7)
Hematologic response at transplant, *n*; % First or second complete remission Marrow aplasia	34 (79) 9 (21)
FLT3/ITD mutation at transplant, *n*; % Negative Positive	27 (63) 16 (27)
Time from diagnosis to transplant in months; median, range	16.7 (5.1–76.0)

### Transplant data

#### Baseline characteristics of the transplanted patients

Twelve patients were transplanted from HLA-matched sibling, and 30 patients received either 10/10 HLA-matched unrelated donor (*n* = 22) or 9/10 HLA-mismatched grafts (*n* = 8). One patient underwent haploidentical transplantation from sister. Peripheral blood was a source of stem cells for all transplanted patients. In total, 26 patients received myeloablative conditioning (MAC), whereas reduced-intensity conditioning (RIC) was provided for 17 subjects. MAC consisted of busulfan and cyclophosphamide (BuCy), and fludarabine-based regimens were given as RIC. Anti-thymocyte globulin (ATG) was administered for all patients who had received a graft from the unrelated donors. Graft versus host disease (GHVD) prophylaxis included cyclosporine and methotrexate in all but one patient with haploidentical transplantation who received cyclosporine with mycophenolate mofetil and post-transplant cyclophosphamide.

#### Outcome of the transplanted patients

There were no primary graft failures (PGF). Median time from transplantation to acute graft versus host disease (GVHD) was 17 days (range 7–106). Acute and chronic GVHD developed in 58% and 14% of patients, respectively. Acute GVHD grade III/IV was present in 6 patients. Two patients had limited and 4 extensive chronic GVHD.

Twelve patients demonstrated mucositis grade 3 or 4 after transplantation. No other severe infectious complications were demonstrated. Two subjects developed post-cyclophosphamide hemorrhagic cystitis, one patient had veno-occlusive disease and one had acute renal failure. Six patients had CMV reactivation prior to day + 30. No patients died within the first 30 days, whereas 6 patients expired prior to day + 100 after transplantation: 3 due to early leukemia progression and subsequent chemo-resistance, 2 as a consequence of steroid-resistant acute GVHD, and 1 patient for aspergillosis.

Post-allograft CR rate amounted to 88% including 4 patients who were transplanted in MA and converted to CR. Five patients who were transplanted in MA had leukemia progression. In total, among 9 patients transplanted in MA, 4 patients were alive at last contact. Twenty-eight (65%) patients achieved *FLT3/ITD* negativity at day + 30 after Allo-SCT, and 21 of them are still alive. In contrast, only 1 patient is alive among those who were FLT3-mutated at day + 30. Non-relapse mortality (NRM) at 2 years was 7%.

In total, 17 patients relapsed after median of 4.8 months (range 0.9–27.4) following transplantation. We look at different variables which may have an impact on the incidence of relapse. The following factors were analyzed: hematological status at transplant, donor source, type of conditioning, the occurrence of acute GVHD, and the presence of *FLT3/ITD* mutation at transplantation and at day + 30 after procedure. The relapse incidence (RI) was lower for patients who were FLT3 MRD negative at transplant when compared with those who remained FLT3 MRD positive (41% vs 59%; *r* = 0.36; *p* = 0.01). The patients who eradicated *FLT3/ITD* mutation at day + 30 post-transplantation had significantly lower RI than those who had detectable *FLT3/ITD* (23% vs 76%; *r* = 0.7; *p* < 0.001). No other correlations were found. Five patients who were FLT3-mutated at transplant eradicated this mutation after allografting. Four patients are alive and remain in CR, and one patient died due to severe GVHD with subsequent infectious complications. Of note is that the latter one had no features of leukemia relapse. Moreover, a strong positive correlation between the post-transplant *FLT3/ITD* status and survival was demonstrated (*r* = 0.65; *p* < 0.001).

NPM1 mutation at diagnosis was tested in 22 AML patients and was detectable in 10 (45%). There were 7 post-transplant relapses in FLT3 MRD(+)/NPM(+) group, and 2 subjects had leukemia recurrence in FLT3 MRD(+)/NPM(−) group (*p* = 0.62). There was one leukemia recurrence in FLT3 MRD(−)/NPM(−) group.

Data on MRD measured by flow cytometry (MRD-FC) at transplant were available in 28 patients, and positive results were demonstrated in 21. There was a strong positive correlation between FLT3 MRD(+) and MRD-FC(+) (*r* = 0.53; *p* = 0.03).

There were 8/13 (62%) post-transplant leukemia relapses in patients who were FLT3 MRD(+)/MRD-FC(+), 2/8 (25%) relapses in FLT3 MRD(−)/MRD-FC (+,) group and 1/7 (14%) disease recurrence in those who had FLT3 MRD(+)/MRD-FC(−).

At the last follow-up, 21 (49%) patients died. The main causes of death included disease progression (*n* = 15), infectious complications (*n* = 2), and steroid-resistant GVHD (*n* = 2). In 2 cases, the cause of death remained unknown. In total, there were 18 (86%) deaths within the first 2 years after transplant.

Twenty-wo patients (51%) are alive at the last contact, and 21 remain in hematological and molecular CR. All those patients had full donor chimerism. One patient had leukemia relapse and received salvage chemotherapy. Median follow-up from diagnosis and from transplantation was 2.1 years (range 0.6–10.9) and 15 months (range 1–119), respectively. Median follow-up for those who are alive after procedure is 34.1 month (range 1.0–119.6). Transplant data are summarized in Table 2.

**Table 2:** Transplant data

Variable	*n* = 43
Type of donor, *n*;% Related 10/10-HLA matched unrelated 9/10-HLA-matched unrelated haploidentical	12 (28) 22 (51) 8 (19) 1 (2)
Myeloablative conditioning, *n*; %	26 (60)
Conditioning regimen, n; % Busulfan/cyclophosphamide Treosulfan/fludarabine Busulfan/fludarabine Fludarabine/TBI	26 (60) 10 (23) 3 (7) 4 (10)
Number of transplanted CD34-positive cells (× 10^6^/kg); median, range	5.4 (3.1–20.4)
ANC > 0.5 (× 10^9^/L); median, range	16 (11–29)
PLT > 20 (× 10^9^/L); median, range	15 (10–32)
Acute GVHD, n; %	12 (58)
Acute GVHD III/IV, n; %	6 (14)
Chronic GVHD, n; %	6 (14)
Hematologic relapse, n; %	17 (39)
Alive at last contact, n; %	22 (51)
FLT3/ITD mutation at day + 30, *n*; % Negative Positive	28 (65) 15 (35)
Median follow-up from transplantation, months; median, range	15 (1–119)

*ANC* absolute neutrophil count; *GVHD* graft versus host disease; *PLT* platelet count; *TBI* total body irradiation

Patients who relapsed after transplantation received a variety of salvage treatments depending on whether they were fit or frail. In total, 11 patients were eligible for intensive chemotherapy and they received CLAM (*n* = 4), COAP (cyclophosphamide, vincristine, cytarabine, prednisone; *n* = 3), high-dose cytarabine (*n* = 2), DAC (*n* = 1), and multi-kinase inhibitor—lestaurtinib (*n* = 1). Two patients underwent second allogeneic transplantation following intensive chemotherapy. Six patients received palliative treatments which are the following: hydroxyurea (*n* = 3) and low-doses cytarabine (*n* = 3). None of the patients received midostaurin and any other FLT3-inhibitor as maintenance.

**Fig. 1:**
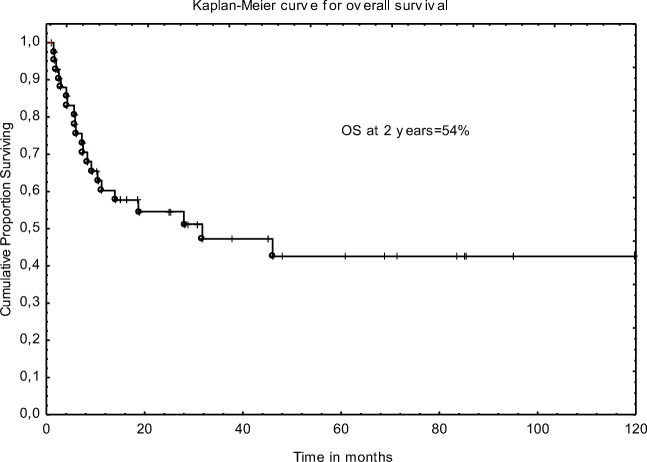
Overall survival for FLT3-mutated AML patients

**Fig. 2:**
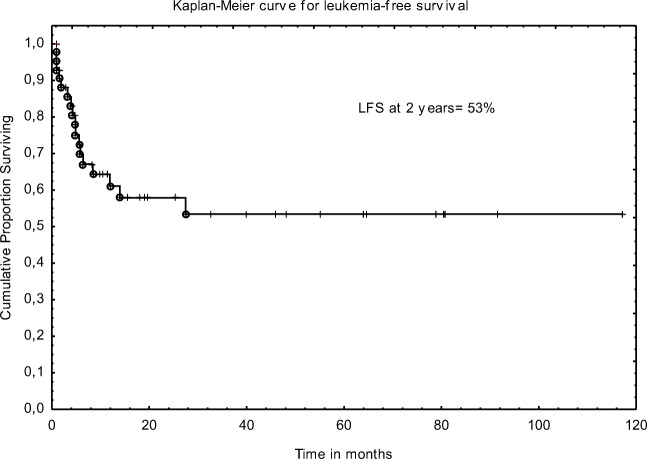
Leukemia-free survival for FLT3-mutated AML patients

The 2-year LFS and OS were 53% and 54%, respectively. Figures 1 and 2.Median OS from diagnosis and from Allo-SCT were 3.2 years and 28 months. Median LFS after Allo-SCT was 27 months. There was no statistical difference in OS between patients transplanted in CR1/2 and MA. Estimated OS at 2 years were 59% and 33%; (*p* = 0.17), respectively. In univariate analysis, unrelated donor, undetectable FLT3/ITD mutation, and normal liver size before transplant were found to influence LFS; however, only FLT3 status remained significance in multivariate analysis (HR 5.3 95%CI 1.97–14.2); *p* < 0.001). The following factors influenced the OS in multivariate analysis: type of donor and mutational status. Details are presented in Tables 3 and 4.

**Table 3:** Univariate and multivariate analysis of risk factors for overall survival

Univariate analysis (log rank)			Multivariate analysis (Cox regression)	
Risk factor	OS at 2 years	*P* value	HR (95% CI)	*P* value
*Type of donor*				
Related *n*=12	19%	0.01	0.35 (0.12-1.0)	0.05
Unrelated *n*=31	68%			
*FLT3/ITD status at transplant*				
Negative *n*=27	74%	0.001	2.67 (0.94-7.56)	0.06
Positive *n*=16	21%

**Table 4:** Univariate and multivariate analysis of risk factors for leukemia-free survival

Univariate analysis (log rank)			Multivariate analysis (Cox regression)	
Risk factor	LFS at 2 years	*P* value	HR (95%CI)	*P* value
*FLT3/ITD status at transplant*				
Negative *n*=27	74%	0.001	5.3 (1.97-14.2)	< 0.001
Positive *n*=16	25%			
*Type of donor*				
Unrelated *n*=31	67%	0.02	-	
Related *n*=12	28%			
*Hepatomegaly at diagnosis*				
No *n*=36	61%	0.09	-	
Yes *n*=7	38%			

## Discussion

**Fig. 3:**
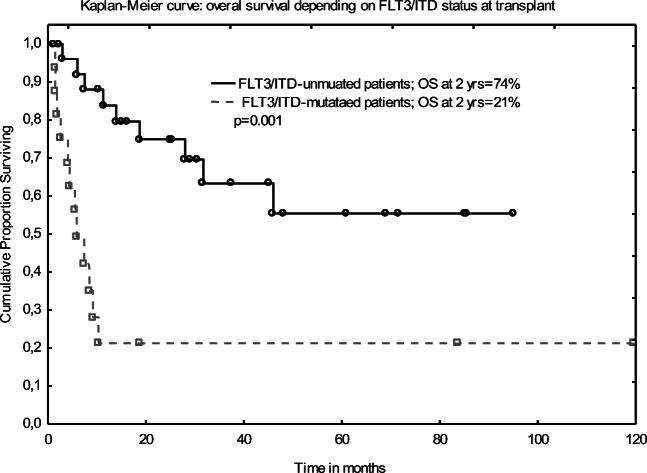
Overall survival depending on FLT3 MRD status at transplant

**Fig. 4:**
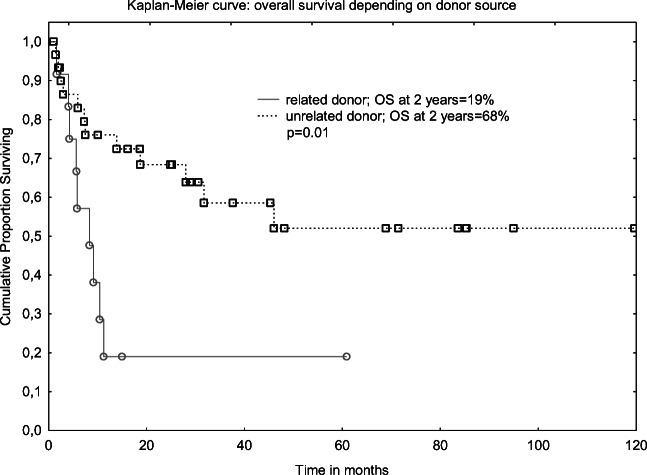
Overall survival depending on donor source

Beneficial role of Allo-SCT in FLT3-mutated AML patients in CR1 remains unclear as these patients had poor prognosis even after procedure. Of note is that the presented data are limited by selection bias and the small number of included patients [7, 8]. To light up this controversy, a systemic review of 9 studies with 772 FLT3-mutated AML patients has been performed. This meta-analysis has demonstrated that SCT (autologous and allogeneic were lumped together) when compared with chemotherapy alone reduced the relapse rate and prolonged OS and LFS. Unexpectedly, there was no advantage of Allo-SCT over Auto-SCT in terms of OS and LFS, and this probably resulted from the higher transplant-related mortality and lower relapse rate (RR) for patients who received allograft. Higher RR for auto-transplanted patients should be explained by no “graft *versus* leukemia” effect. An interesting observation is that some FLT3-mutated AML patients with no suitable donor may benefit from the Auto-SCT. Of note is that these conclusions were based on 3 studies only, and the results were limited by selection bias, the number of included patients, and inclusion of retrospective and non-randomized studies [12] (Figs. 3 and 4).

In contrast, there are several studies showing that Allo-SCT improves outcome in FLT3-mutated AML when performed in CR1. The median LFS in FLT3/ITD-transplanted AML population was 54.1 month when compared with 8.6 months for FLT3/ITD-non-transplanted group (*p* = 0.03) [13]. The comparable parameters for our cohort were 27 and 3 months, respectively [data not published].The FLT3-mutated patients who received Allo-SCT as consolidation at CR1 had longer OS and relapse-free survival (RFS) than patients left on chemotherapy alone. OS at 3 year were 54% and 24%, respectively. Multivariate analysis showed the advantage of Allo-SCT over chemotherapy irrespective of *FLT3/ITD* allelic ratio [14]. The results of transplantation for FLT3-mutated AML beyond CR1 are less encouraging. The post-transplant outcome of 200 FLT3-mutated AML patients was presented by MD Anderson Group. The patients were transplanted both in CR1 and beyond, including those with active disease. Median follow-up was 27 months, and OS and LFS for the entire study cohort at 2 years were 43% and 41%, respectively. RR was the lowest for those who were transplanted in CR1/CR2 and with FLT3 MRD negativity. The worst outcome was demonstrated for patients transplanted in active disease; 85% of them had relapse. In multivariate analysis, hematological/morphological remission and undetectable FLT3 MRD at transplant were found to improve outcome [9].

Median follow-up for our cohort was 15 months, and OS and LFS at 2 years were found to be slightly better than those reported in Gaballa study [9]—54% and 53%, respectively. Of note is that disease status had no impact on the post-transplant outcome. OS at 2 years did not differ between patients transplanted in CR1/2 or MA: 59% and 33%, respectively. Patients who had pre-transplant CR1/2 and FLT3 MRD negativity had the excellent 2-year OS (80%). We have also demonstrated that FLT3 MRD negativity is a strong predictor of OS and LFS in multivariate analysis. The 2-year OS for FLT3 MRD negative and FLT3 MRD positive groups at transplant was 74% and 21%, respectively. Our results strongly suggest that pre-transplant detection of *FLT3/ITD* mutation by PCR may serve as a reliable MRD marker which predicts post-transplant outcome better than the hematological disease status. Data on the influence of pre-transplant *FLT3/ITD* status on outcome after allogeneic transplantation are scarce and actually limited to Gaballa study [9]. Our results are in line with this report, despite lower number of recruited patients. Nevertheless, further studies with larger number of patients are needed to draw final conclusions.

In contrary to previously mentioned report [9], it was demonstrated that *FLT3/ITD* mutation may occur at any time during leukemia course, and this mutation should not be used as MRD marker in AML [15]. The introduction of more advanced PCR techniques may increase the sensitivity of PCR method and therefore early therapeutic intervention [16]. Moreover, it would be valuable to correlate different MRD methods which are the following: PCR assay for FLT3/ITD and multi-parameter flow cytometry (FC).The results of MRD by flow cytometry (MRD-FC) at transplant were available in 28 patients from our cohort, and MRD-FC was positive in 21. There was a strong positive correlation between pre-transplant FLT3 MRD(+) and MRD-FC(+). Data on the role of pre-transplant MRD measured by FC in FLT3-mutated patients have been published recently. Only MRD-FC-positive patients transplanted from HLA-matched sibling donor were found to have poor outcome when compared with MRD-FC-negative transplants from both sibling and haploidentical donors. Interestingly, the outcome of haplo-SCT for MRD-FC-positive patients was comparable with that demonstrated for subjects with pre-transplant MRD-FC-negativity [17]. In the light of the recent findings and when confirmed in prospective studies, it seems reasonable to consider FLT3 inhibitor as a part of induction therapy and post-transplant maintenance [18, 19]. A double-blind randomized study showed that the addition of midostaurin to standard chemotherapy significantly improved overall and event-free survival in FLT3-positive AML patients; however, midostaurin was not beneficial as maintenance treatment after transplantation [18]. In the phase II RADIUS study, FLT3-positive AML patients who underwent Allo-SCT in CR1 were randomized to receive standard of care (SOC) with or without midostaurin. No differences in relapse-free survivals (RFS) between arms at 18 months and 24 months post-transplantation were demonstrated [20]. In contrast, post-transplant sorafenib improved RFS and OS when compared with placebo in the SORMAIN trial [21].

Returning to our cohort, patients who received a graft from unrelated donor fared much better than those transplanted from sibling. The latter stands in contrast with Gaballa study [9], in which unrelated donor was a predictor of worse outcome (LFS/NRM). Our findings are difficult to explain, especially in the context of recent studies which showed the comparable results of Allo-SCT irrespective of donor source [22]. There was no impact of pre-transplant conditioning on the results of transplantation for FLT3-mutated AML, both in MD Anderson [9] and in our study; however, other groups found MAC to be more effective than RIC in terms of LFS [23]. It was also demonstrated that older age (> 60 years) was not associated with the worse outcome in FLT3-mutated AML patients undergoing Allo-SCT. This procedure was found to be feasible with RIC, and CR1 at transplant was a predictor of favorable outcome [24]. There were only 4 elderly patients in our cohort and 3 of them are alive; however, the follow-up is relatively short (11 months).

In conclusion, patients with FLT3-mutated AML should proceed to allogeneic stem cell transplantation as soon as possible in the disease course. Most promising results are associated with the FLT3 MRD eradication before transplantation. Post-transplant maintenance with FLT3 inhibitors may increase survival.

## References

[1] Stirewalt DL, Radich JP (2003). The role of FLT3 in hematopoietic malignancies. Nat Rev Cancer.

[2] Frӧhling S, Schlenk RF, Breitruck J (2002). Prognostic significance of activating FLT3 mutations in younger adults (16 to 60 years) with acute myeloid leukemia and normal cytogenetics: a study of the AML study group Ulm. Blood..

[3] Rombouts WJ, Blokland I, Lowenberg B (2000). Biological characteristics and prognosis of adult acute myeloid leukemia with internal tandem duplications in the Flt3 gene. Leukemia..

[4] Dӧhner H, Estey E, Grimwade D (2017). Diagnosis and management of AML in adults: 2017 ELN recommendations from an international expert panel. Blood..

[5] Bornhauser M, Illmer T, Schaich M (2007). Improved outcome after stem-cell transplantation in FLT3-ITD positive AML. Blood..

[6] Lin PH, Lin CC, Yang HI, Li LY, Bai LY, Chiu CF, Liao YM, Lin CY, Hsieh CY, Lin CY, Ho CM, Yang SF, Peng CT, Tsai FJ, Yeh SP (2013). Prognostic impact of allogeneic hematopoietic stem cell transplantation for acute myeloid leukemia patients with internal tandem duplication of FLT3. Leuk Res.

[7] Gale RE, Hills R, Kottaridis PD (2005). No evidence that FLT3 status should be considered as an indicator for transplantation in acute myeloid leukemia (AML): an analysis of 1135 patients, excluding acute promyelocytic leukemia, from the UK MRC AML10 and 12 trials. Blood..

[8] Brunet S, Labopin M, Esteve J, Cornelissen J, Socié G, Iori AP, Verdonck LF, Volin L, Gratwohl A, Sierra J, Mohty M, Rocha V (2012). Impact of FLT3 internal tandem duplication on the outcome of related and unrelated hematopoietic transplantation for adult acute myeloid leukemia in first remission: a retrospective analysis. J Clin Oncol.

[9] Gaballa S, Saliba R, Oran B, Brammer JE, Chen J, Rondon G, Alousi AM, Kebriaei P, Marin D, Popat UR, Andersson BS, Shpall EJ, Jabbour E, Daver N, Andreeff M, Ravandi F, Cortes J, Patel K, Champlin RE, Ciurea SO (2017). Relapse risk and survival in patients with FLT3 mutated acute myeloid leukemia undergoing stem cell transplantation. Am J Hematol.

[10] Huang Q, Chen W, Gaal KK et al A rapid, one step assay for simultaneous detection of *FLT3/ITD* and *NPM1* mutations in AML with normal cytogenetics. Br J Haematol 142:489–492 10.1111/j.1365-2141.2008.07205.x 18477048

[11] Dohner H, Estey EH, Amadori S (2010). Diagnosis and management of acute myeloid leukemia in adults: recommendations from an international expert panel, on behalf of the European LeukemiaNet. Blood..

[12] Ma Y, Wu Y, Shen Z (2015). Is allogeneic transplantation really the best treatment for FLT3/ITD-positive acute myeloid leukemia? A systemic review. Clin Transpl.

[13] DeZern AE, Sung A, Kim S, Smith BD, Karp JE, Gore SD, Jones RJ, Fuchs E, Luznik L, McDevitt M, Levis M (2011). Role of allogeneic transplantation for FLT3/ITD acute myeloid leukemia: outcomes from 133 consecutive newly-diagnosed patients from a single institution. Biol Blood Marrow Transplant.

[14] Oran B, Cortes J, Beitinjaneh A, Chen HC, de Lima M, Patel K, Ravandi F, Wang X, Brandt M, Andersson BS, Ciurea S, Santos FP, de Padua Silva L, Shpall EJ, Champlin RE, Kantarjian H, Borthakur G (2016). Allogeneic transplantation in first remission improves outcomes irrespective of FLT3-ITD allelic ratio in FLT3-ITD-positive acute myelogenous leukemia. Biol Blood Marrow Transplant.

[15] Nazha A, Cortes J, Faderl S, Pierce S, Daver N, Kadia T, Borthakur G, Luthra R, Kantarjian H, Ravandi F (2012). Activating internal tandem duplication mutations of the fms-like tyrosine kinase-3 (FLT3-ITD) at complete remission and relapse in patients with acute myeloid leukemia. Haematologica..

[16] Grunwald MR, Tseng LH, Lin MT (2004). Improved FLT3/ITD PCR assay predicts outcome following allogenic transplant for AML. Biol Blood Marrow Transplant.

[17] Zhao X, Wang Z, Ruan G, Liu Y, Wang Y, Zhang X, Xu L, Huang X, Chang Y (2018). Impact of pre-transplantation minimal residual disease determined by multiparameter flow cytometry on the outcome of AML patients with FLT3-ITD after allogeneic stem cell transplantation. Ann Hematol.

[18] Stone RM, Mandrekar SJ, Sanford BL, Laumann K, Geyer S, Bloomfield CD, Thiede C, Prior TW, Döhner K, Marcucci G, Lo-Coco F, Klisovic RB, Wei A, Sierra J, Sanz MA, Brandwein JM, de Witte T, Niederwieser D, Appelbaum FR, Medeiros BC, Tallman MS, Krauter J, Schlenk RF, Ganser A, Serve H, Ehninger G, Amadori S, Larson RA, Döhner H (2017). Midostaurin plus chemotherapy for acute myeloid leukemia with a FLT3 mutation. N Engl J Med.

[19] Bazarbachi A, Labopin M, Battipaglia G (2019). Allogeneic stem cell transplantation for FLT3-mutated acute myeloid leukemia: *in vivo* T-cell depletion and posttransplant sorafenib maintenance improve survival. A retrospective acute leukemia working party- European Society for Blood and Marrow Transplant Study. Clin Hematol Int.

[20] Maziarz RT, Patnaik MM, Scott BL, et al. (2018) Radius: a phase 2 randomized trial investigating standard of care +/− midostaurin after allogeneic stem cell transplantation in FLT3/ITD-mutated AML. 60^th^ ASH Annual Meeting and Exposition, San Diego, CA. Abstract 633

[21] Burchert A, Bug G, Finke J, et al. (2018) Sorafenib as maintenance therapy post allogeneic stem cell transplantation for FLT3-ITD positive AML: results from the randomized, double-blind, placebo-controlled multicenter Sormain Trial. . 60^th^ ASH Annual Meeting and Exposition, San Diego, CA. Abstract 661

[22] Di Stasi A, Milton DR, Poon LM (2014). Similar transplantation outcome for acute myeloid leukemia and myelodysplastic syndrome patients with haploidentical versus 10/10 human leukocyte antigen-matched unrelated and related donors. Biol Blood Marrow Transplant.

[23] Fleischmann M, Schnetzke U, Schrenk KG, Schmidt V, Sayer HG, Hilgendorf I, Hochhaus A, Scholl S (2017). Outcome of FLT3-ITD-positive acute myeloid leukemia: impact of allogeneic stem cell transplantation and tyrosine kinase inhibitor treatment. J Cancer Res Clin Oncol.

[24] Poire X, Labopin M, Polge E (2018). Allogeneic stem cell transplantation benefits for patients ≥60 years with acute myeloid leukemia and FLT3 internal tandem duplication: a study from the acute leukemia working Party of the European Society for blood and marrow transplantation. Haematologica..

